# Therapeutic reprogramming of natural killer cells for solid tumors: expanding frontiers into rare and ultra-rare malignancies

**DOI:** 10.3389/fmolb.2026.1955532

**Published:** 2026-09-23

**Authors:** Francesca Rosato, James W. Hodge

**Affiliations:** Center for Immuno-Oncology, Center for Cancer Research, National Cancer Institute, National Institutes of Health, Bethesda, MD, United States

**Keywords:** adoptive cell therapy, chimeric antigen receptor, clinical trials, immunotherapy, natural killer cells, rare tumors, solid tumors

## Abstract

Rare and ultra-rare solid tumors collectively account for approximately one-quarter of all cancer diagnoses yet remain associated with poor clinical outcomes. Delayed diagnosis, limited biological characterization, few therapeutic options, and the logistical challenges of conducting adequately powered clinical trials underscore the potential for adaptable cellular immunotherapies that can be applied across diverse tumor histologies. Natural killer (NK) cells have emerged as a promising platform for adoptive cell therapy because of their ability to recognize and eliminate malignant cells independently of major histocompatibility complex restriction, their favorable safety profile, and their suitability for standardized allogeneic, off-the-shelf manufacturing. This review examines the progression of NK cell-based therapies from their biological and therapeutic foundations in solid tumors to their emerging application in rare and ultra-rare malignancies. Recent advances in NK cell engineering are enabling multifunctional cell products designed to enhance tumor recognition, trafficking, persistence, and resistance to the immunosuppressive tumor microenvironment. We also review emerging clinical evidence demonstrating the feasibility and safety of these approaches while highlighting the major biological and translational barriers that continue to limit durable responses. Building on these advances, attention is given to the unique challenges of rare and ultra-rare cancers, where small patient populations, tumor heterogeneity, limited therapeutic targets, and logistical constraints pose unique challenges to conventional drug and cellular therapy development. Finally, we discuss how scalable off-the-shelf manufacturing, rational combination strategies, innovative clinical trial designs, and next-generation technologies, including multiplex gene editing, armored NK cells, and *in vivo* engineering, may facilitate this translation. Collectively, progress in NK cell therapy across solid tumors provides a framework for extending increasingly adaptable and scalable immunotherapeutic platforms to rare and ultra-rare malignancies, with the potential to expand treatment opportunities for patient populations that have historically had limited therapeutic options.

## 1 Introduction

Rare and ultra-rare solid cancers, although individually uncommon, collectively account for approximately 20%–25% of all cancer diagnoses and represent a substantial unmet clinical need. Their low incidence poses significant challenges to therapeutic development, including delayed diagnosis, limited biological and molecular characterization, and difficulties in conducting adequately powered prospective clinical trials (Botta et al., 2020; Gatta et al., 2011). Consequently, treatment strategies are frequently extrapolated from more common malignancies despite important biological differences, leaving many patients facing significantly lower survival rates compared to those with more common cancers. These limitations underscore the need for innovative therapeutic platforms that can be broadly adapted across diverse tumor types without relying on disease-specific drug development (Bradford et al., 2016; Casali et al., 2015).

Adoptive cell therapy (ACT) has emerged as one of the most promising immunotherapeutic strategies for harnessing the intrinsic cytotoxicity of immune cells to eliminate malignant cells. While conventional therapies like chemotherapy and radiation remain fundamental, their clinical efficacy is frequently limited by severe systemic toxicities, insufficient tumor targeting, and acquired multidrug resistance (Juthani et al., 2024; Wang and Tepper, 2021). Conversely, ACT exploits the body’s own immune system to enhance immune recognition and control of tumor growth (Rosenberg and Restifo, 2015). ACT encompasses a broad range of cellular platforms derived from both adaptive and innate immunity, including T cells and natural killer (NK) cells. While T cell-based therapies have dominated clinical development and transformed the treatment of hematological malignancies, their translation to solid tumors has been hindered by antigen heterogeneity, lack of immune cell trafficking, persistence, and the highly immunosuppressive tumor microenvironment (TME) (Fousek and Ahmed, 2015). These challenges are particularly amplified in rare and ultra-rare solid tumors, where biological diversity and limited patient numbers further complicate the development of personalized cellular therapies (Sharifnia et al., 2017; van der Graaf et al., 2025).

Considering these obstacles, NK cells represent a highly promising avenue for therapeutic intervention. As key effector cells of the innate immune system, NK cells act as the body’s first line of defense in tumor immunosurveillance and antitumor immune responses by continuously scouting and eliminating malignant cells (Muller et al., 2017; Waldhauer and Steinle, 2008). Accordingly, NK cell-based ACT has emerged as a promising alternative to T cell-based therapies for cancer immunotherapy. Unlike T cells, NK cells retain the ability to target tumors that evade adaptive immune responses through major histocompatibility complex (MHC) downregulation. Moreover, NK cell-based therapies are associated with a lower risk of severe immune-mediated toxicities such as cytokine release syndrome (CRS) and immune effector cell-associated neurotoxicity syndrome (ICANS) (Dagher and Posey, 2023; Liu et al., 2020). Beyond efficacy, safety and logistics in terms of handling these cells have attracted a great deal of attention. NK cell therapies can be manufactured from healthy donors as standardized allogeneic, off-the-shelf products, providing a scalable and readily deployable platform that is particularly well suited to rare cancers, where individualized autologous manufacturing and disease-specific therapeutic development are often economically and logistically impractical (Fabian and Hodge, 2021; Shi et al., 2024).

Recent advances in genetic engineering, cell manufacturing, and synthetic immunology have further expanded the therapeutic potential of NK cells. Engineered NK cell platforms—including chimeric antigen receptor (CAR)-NK cells, cytokine-armored NK cells, memory-like (ML) NK cells, and engager-based strategies—are being developed to enhance persistence, trafficking, metabolic fitness, and antitumor activity while maintaining the favorable safety profile that distinguishes NK cell therapies from many T cell-based approaches (Balkhi et al., 2025; Page et al., 2024). Collectively, these advances position NK cell-based ACT as a promising therapeutic platform capable of addressing both the biological barriers of solid tumors and the translational challenges inherent to rare and ultra-rare malignancies.

This review discusses the biological rationale for NK cell-based adoptive immunotherapy in solid tumors, including rare and ultra-rare tumors, and summarizes the current clinical landscape of these emerging therapies. We highlight recent advances in NK cell engineering and engager-based approaches designed to improve tumor specificity, persistence, and function within the hostile TME. Finally, we discuss the major translational challenges that remain, including immune evasion, manufacturing scalability, and clinical implementation, as well as emerging strategies that may enable broader adoption of NK cell therapies for patients with rare and ultra-rare solid cancers.

## 2 Mechanisms of NK cell-mediated antitumor immunity

NK cells serve as critical innate effectors in immune surveillance against virally infected and malignant cells. They account for 5%–20% of circulating peripheral blood mononuclear cells (PBMCs) and exert MHC-unrestricted cytotoxicity, without prior antigen exposure (Meza Guzman et al., 2020). NK are classified into two distinct subsets based on the relative expression of the surface markers CD56 and CD16. The CD56^bright^ CD16^low/-^ subset contributes to cytokine production and is primarily immunomodulatory, while CD56^dim^CD16^+^ NK cells exhibit robust cytotoxic activity, similar to that of CD8^+^ T cells but without a rearranged T cell receptor (TCR) complex (Abel et al., 2018; Freud et al., 2017).

### 2.1 NK cell activation and the “missing-self” paradigm

Unlike T cells, which recognize peptide antigens presented by MHC molecules through clonotypic TCRs, NK cell activation relies on a dynamic balance between germline-encoded, competing inhibitory and activating signals. The cumulative signals from these opposing inputs determine whether NK cells engage in a “kill” or “spare” response (Lanier, 2008; Sun and Lanier, 2011). Healthy cells are preserved because they express MHC class I molecules which bind inhibitory killer cell immunoglobulin-like receptors (KIRs) that deliver inhibitory signals preventing NK cell activation. Conversely, NK cells proceed to eliminate cells that downregulate MHC class I molecules on their surface, through the mechanism of missing-self recognition (Raulet, 2006). Transformed cells frequently upregulate stress-induced ligands recognized by activating receptors such as NKG2D, DNAM-1, and the natural cytotoxicity receptors (NKp30, NKp44, and NKp46), while simultaneously downregulating MHC class I expression (Long et al., 2013). This shift in receptor signaling, described by the concepts of “missing-self” and “induced-self” recognition, licenses NK cells to selectively eliminate malignant targets while sparing healthy cells (Brodin et al., 2009; Joncker et al., 2009; Joncker et al., 2010; Laskowski et al., 2022).

### 2.2 Effector mechanisms of tumor cytotoxicity

Once activated, NK cells eradicate tumor cells by a variety of mechanisms that ultimately result in the direct lysis or triggered apoptosis of target cells. These include the i) release of cytolytic granules loaded with granzymes and pore-forming proteins, ii) upregulation of death ligands, including Fas ligand (FasL) and tumor necrosis factor (TNF)-related apoptosis-inducing ligand (TRAIL), iii) release of cytokines such as TNFα, interferon gamma (IFN-γ), granulocyte-macrophage colony-stimulating factor (GM-CSF), and C-C motif chemokine ligand 5 (CCL5)—which promote dendritic cell maturation, enhance macrophage polarization, recruit additional immune effectors, and orchestrate adaptive antitumor immunity. Finally, iv) expression of the Fc receptor CD16 enables NK cells to mediate antibody-dependent cellular cytotoxicity (ADCC), whereby therapeutic antibodies bridge NK cells to antibody-coated tumor cells, representing a major mechanism of action for several clinically approved monoclonal antibody therapies (Bryceson et al., 2005; Fauriat et al., 2010; Takeda et al., 2001). Despite these potent cytotoxic mechanisms, NK cells often fail to eradicate established solid tumors because the TME progressively suppresses their function.

### 2.3 Immune evasion in the TME

NK cell activity against solid tumors is profoundly impaired by the hostile and immunosuppressive TME. Rapidly dividing cancer cells outcompete immune cells for essential nutrients, creating a state of metabolic starvation. The TME is also hypoxic and highly acidic due to lactic acid accumulation, impairing the bioenergetic fitness of immune cells. Tumor cells, stromal cells, and infiltrating immunosuppressive populations, including regulatory T cells (Tregs), myeloid-derived suppressor cells (MDSCs), tumor-associated macrophages (TAMs), and cancer-associated fibroblasts (CAFs), collectively establish an environment that impairs NK cell infiltration and activation (Melaiu et al., 2019; Terren et al., 2019; Vitale et al., 2014). Furthermore, many cancer-associated soluble mediators are responsible for NK cell dysfunction within the TME, most notably transforming growth factor-beta (TGF-β), interleukin-10 (IL-10), indoleamine 2,3-dioxygenase (IDO), and prostaglandin E2 (PGE2). These cytokines contribute to suppressing NK cell proliferation and cytotoxicity by directly downregulating the expression of key activating receptors. Concurrently, chronic exposure to tumor-derived stress ligands and persistent inflammatory signaling induces the upregulation of inhibitory checkpoint receptors on NK cell surface (Batlle and Massague, 2019; Tumino et al., 2023; Zaiatz-Bittencourt et al., 2018).

These barriers are equally, if not more, relevant in rare and ultra-rare solid tumors, many of which are characterized by dense stromal architecture, low immune infiltration, and poorly defined immunobiology. Consequently, strategies that enhance NK cell function, trafficking, and resistance to TME-mediated suppression are critical for therapeutic success in these orphan malignancies. Accordingly, next-generation NK cell therapies seek to enhance activation, persistence, metabolic fitness, and resistance to TME-mediated suppression through cytokine priming, *ex vivo* expansion, receptor engineering, and genetic modification (Myers and Miller, 2021).

## 3 NK cell-based therapeutic platforms

To overcome tumor-induced immunosuppression, NK cell-based therapeutic intervention primarily focuses on three main approaches: ACT, cytokine-based therapies, and antibody-directed therapies. These strategies either expand and enhance the functional activity of NK cells or stimulate endogenous NK cell responses through immunomodulatory mechanisms and are increasingly being integrated into combination regimens to maximize therapeutic benefit.

### 3.1 Adoptive NK cell therapy

ACT is the most extensively investigated NK cell-based strategy for clinical intervention. It involves the administration of *ex vivo* expanded or genetically engineered NK cells into patients. Most current NK cell platforms have evolved in response to the limitations of earlier-generation approaches. Depending on the cellular source and engineering strategy, ACT encompasses several platforms, including autologous NK cells, allogeneic donor-derived NK cells, umbilical cord blood (CB)-derived NK cells, induced pluripotent stem cell (iPSC)-derived NK cells, immortalized cell lines, and CAR-NK cells (Figure 1) (Franks et al., 2020; Shimasaki et al., 2020; Vivier et al., 2024). Among these, allogeneic and iPSC-derived NK cells have become increasingly prominent because they enable standardized, large-scale off-the-shelf manufacturing with reduced production costs and rapid availability for clinical use owing to their minimal risk of graft-versus-host disease (GVHD).

**FIGURE 1:**
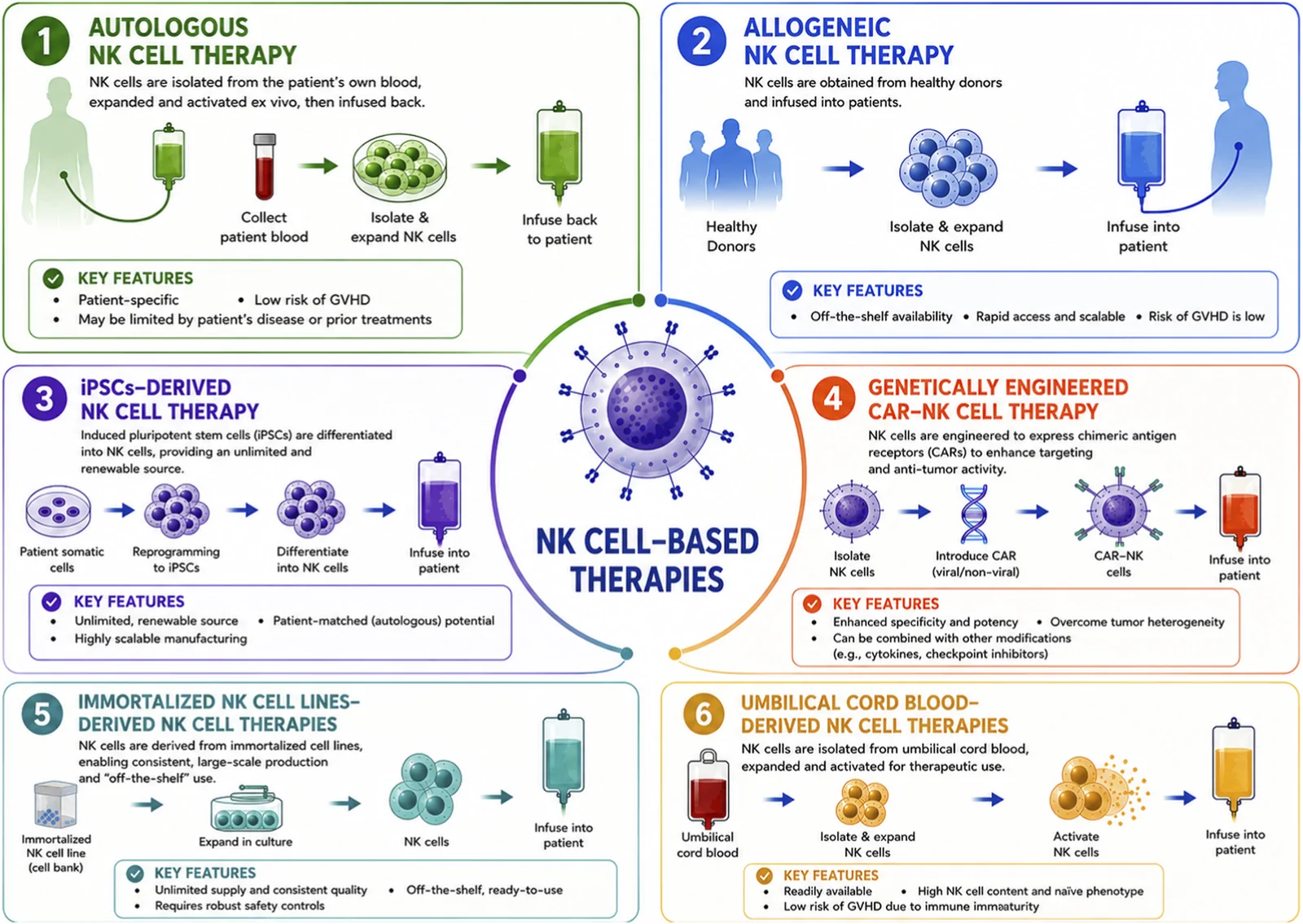
Overview of NK cell-based therapeutic platforms for cancer immunotherapy. Schematic representation of the major natural killer (NK) cell-based therapeutic strategies currently under investigation and clinical development. NK immunotherapies can be generated from multiple cellular sources, which include (1) autologous NK cell therapy, in which patient-derived NK cells are isolated, expanded, and activated *ex vivo* before reinfusion. (2) Allogeneic NK cell therapy using NK cells obtained from healthy donors, enabling off-the-shelf treatment approaches with broader clinical accessibility. (3) Induced pluripotent stem cell (iPSC)-derived NK cell therapy, a renewable and highly scalable source of homogeneous NK cells suitable for standardized manufacturing. (4) Genetically engineered chimeric antigen receptor (CAR)-NK cell therapy, in which NK cells are modified with synthetic antigen receptors to enhance tumor-specific recognition and cytotoxicity. (5) Immortalized NK cell line-derived therapies, exemplified by the NK-92 cell line, which enable standardized and large-scale production of readily available therapeutic products. (6) Umbilical cord blood-derived NK cell therapy, utilizing the highly abundant NK cell progenitors in cord blood to generate functional NK cell products with robust expansion potential. Each platform offers distinct advantages and limitations with respect to cell source, manufacturing scalability, persistence, safety, and clinical applicability, collectively expanding the therapeutic landscape of NK cell-based immunotherapy. GVHD, graft-versus-host disease.

#### 3.1.1 Autologous NK cell therapy

Autologous NK cell therapy represents the earliest form of adoptive NK cell immunotherapy and requires isolation, *ex vivo* expansion, activation, and reinfusion of a patient’s own NK cells. Autologous NK cells are generally expanded using cytokines such as IL-2, IL-15, IL-21, or feeder-cell systems to enhance their proliferation and cytotoxicity before reinfusion (de Rham et al., 2007) and widely demonstrated *in vitro* and *in vivo* cytotoxicity against tumor cell lines. Despite these advantages, however, clinical responses have generally been limited, especially in patients with advanced solid malignancies (Cheng et al., 2013; Parkhurst et al., 2011). One major limitation is likely the recognition of self-human leukocyte antigen (HLA) molecules on tumor cells by these autologous NK cells, which suppress cytolytic activity against the tumor. In solid tumors, the immunosuppressive TME further limits NK cell trafficking, activation and persistence, resulting in poor clinical outcomes. Furthermore, autologous NK cells harvested from heavily pretreated cancer patients often exhibit baseline functional exhaustion and diminished proliferative capacity, limiting their ability to overcome the TME (Myers and Miller, 2021; Wolf et al., 2023). Consequently, although autologous NK cell therapy has an excellent safety profile, its antitumor efficacy is generally inferior to that of donor-derived NK cell approaches.

#### 3.1.2 Allogeneic NK cell therapy

To overcome the limitations of autologous therapy, the field rapidly pivoted allogeneic NK cell therapies consisting of NK cells obtained from healthy donors and infused into patients. There are several tissue sources used for allogeneic NK cells: peripheral blood (PB), CB, or iPSCs. These enable rapid NK cell manufacture to off-the-shelf products following rapid *ex vivo* expansion and/or differentiation, engineering and preservation (Berrien-Elliott et al., 2023; Laskowski et al., 2022). Off-the-shelf NK cell therapies are immediately available, avoiding the laborious weeks for autologous cell production, and have the potential of allowing a single donor-derived product to treat multiple patients.

Compared with autologous NK cells, healthy donor-derived NK cells generally possess superior cytotoxicity, proliferative capacity, and cytokine production, resulting in more potent antitumor activity. The foundational rationale for allogeneic NK cell therapy relies on the “KIR-ligand mismatch” between the donor’s NK cells and the recipient’s HLA class I molecules on tumors. When donor inhibitory KIRs fail to recognize recipient HLA class I molecules, NK cell activation is enhanced through a missing-self response, driving NK cells into a highly activated, cytotoxic state (Ruggeri et al., 2005; Yang et al., 2016). In solid tumors, however, allogeneic NK cells face immunosuppressive barriers imposed by the TME, which restricts tumor infiltration, persistence, and cytotoxic function. Genetic engineering of these cells has focused on improving tumor homing by expressing chemokine receptors that match chemokines secreted by solid tumors. Donor-derived NK cells equipped with CXCR1 or CXCR2 exhibited enhanced migration toward IL-8-producing tumors, whereas CCR7 or CXCR4 expression improved trafficking to CCL19/CCL21-or CXCL12-rich tumor sites, respectively, resulting in greater intratumoral accumulation and improved antitumor responses in preclinical models. In parallel, investigators have enhanced NK cell persistence and functionality by engineering PB-derived NK cells to express membrane-bound or secreted IL-15, enabling prolonged survival and sustained cytotoxicity within the cytokine-deprived TME (Martin et al., 2024; Page et al., 2024).

An important limitation of allogeneic NK cell therapy is that the low risk of GVHD does not eliminate the reciprocal problem of host-versus-graft immune rejection. Recipient T cells can recognize mismatched donor HLA molecules and eliminate infused NK cells, contributing to limited *in vivo* persistence and potentially necessitating lymphodepletion, repeated dosing, or cytokine support. Strategies to reduce rejection include partial HLA matching and genetic engineering to evade host immunity. For example, reducing HLA class I expression through β2-microglobulin (B2M) disruption can limit recognition by host alloreactive T cells but may increase susceptibility to host NK cell “missing-self” responses. Next-generation approaches therefore combine reduced classical HLA expression with nonclassical molecules such as HLA-E, which suppress host NK cells through NKG2A, thereby creating more immune-evasive or “stealth” allogeneic NK cell products (Hoerster et al., 2020). These strategies seek to retain the manufacturing and safety advantages of off-the-shelf NK cell therapy while improving persistence and antitumor activity.

#### 3.1.3 iPSC-derived NK cell therapy

Induced pluripotent stem cells have emerged as the leading source to generate renewable, homogeneous and universal adoptive cell therapy. Unlike primary NK cells isolated from PBMCs or CB, which are subject to substantial donor-to-donor variability, a single genetically engineered iPSC clone can be expanded indefinitely and differentiated into NK cells, enabling the production of a highly standardized, homogeneous, and scalable population of iPSC-derived NK (iNK) cells (Goldenson et al., 2022; Woll et al., 2005). iNK cells express many of the canonical surface markers found on PB-derived NK cells, including CD56, DNAM-1, CD69, NKG2A, NKG2D, and the natural cytotoxicity receptors. However, they typically exhibit lower CD16 expression, which limits their capacity to mediate ADCC.

Recent studies have used CRISPR-Cas9 to disable negative checkpoints, such as the intracellular checkpoint cytokine-inducible SH2-containing protein (CISH) or the inhibitory receptor NKG2A and generate hyperfunctional iNKs. Coupling these genetic modifications with the expression of high-affinity, non-cleavable CD16 to augment ADCC and tumor-specific CARs yields iNK cells with enhanced metabolic fitness and durable serial cytotoxicity in preclinical models of solid tumors (Kumar et al., 2025). The clonal nature of iPSCs, indeed, makes them particularly well suited for multiplex genome engineering, enabling the generation of standardized, genetically defined cell products through clonal selection and unlimited expansion (Elmas et al., 2022; Fisher et al., 2022).

While iPSC-derived NK cells have overcome many of the limitations in terms of scalability, genetic engineering, and standardized manufacturing associated with donor-derived NK cells, their clinical success in solid tumors continues to be constrained by the TME. Recent studies have shifted the focus of iPSC-derived NK cell engineering from simply enhancing cytotoxicity to equipping these cells with the ability to overcome the immunosuppressive barriers in solid tumors. For instance, Fukutani et al. generated genetically engineered human iPSC-derived NK cells expressing CCR2B, CCL19, IL-15, high-affinity CD16, and the NKG2D-DAP10 complex, in a multi-gene approach to simultaneously address several key TME obstacles (Fukutani et al., 2025). Expression of CCR2B improved NK cell migration and infiltration in CCL2-producing tumors, while CCL19 promoted the recruitment of dendritic cells to stimulate local antitumor immune responses. Constitutive IL-15 expression potentiated NK cell survival and persistence in the TME, whereas high-affinity CD16 enhanced ADCC. Additionally, overexpression of the NKG2D-DAP10 signaling complex strengthened NK cell activation and cytotoxicity against tumor cells. Collectively, these modifications resulted in superior tumor infiltration, prolonged persistence, and improved antitumor efficacy in an orthotopic lung cancer model (Fukutani et al., 2025). In a study focused on hepatocellular carcinoma (HCC), Thangaraj et al. engineered iPSC-derived NK cells to resist the immunosuppressive effects of TGF-β within the TME. The investigators genetically disrupted TGF-β receptor 2 (TGFβR2) or introduced dominant-negative TGF-β receptors into iPSC-derived NK cells, thereby preventing TGF-β-mediated signaling. These engineered NK cells maintained their cytotoxic function and cytokine production despite exposure to suppressive levels of TGF-β and demonstrated enhanced antitumor activity in preclinical HCC models (Thangaraj et al., 2024).

#### 3.1.4 Immortalized NK cell lines and CAR-NK cells

Immortalized NK cell lines were developed to overcome many of the manufacturing limitations associated with primary NK cells. Because they proliferate indefinitely *in vitro*, they provide an unlimited supply of homogeneous effector cells from a single master cell bank, eliminating donor-to-donor variability and simplifying large-scale manufacturing. Their stable phenotype also improves batch-to-batch consistency, which is particularly advantageous for off-the-shelf cellular therapies (Fabian and Hodge, 2021; Suck et al., 2016). To date, more than ten immortalized human NK cell lines have been established, with the NK-92 cell line being the only one that has been cleared for clinical investigation (Gong et al., 1994; Hu et al., 2019). The NK-92 cell line (CD3^neg^CD56^bright^) was isolated from the PB of a patient with lymphoma and is a permanent, IL-2-dependent NK cell line. NK-92 cells express the full spectrum of activating receptors, with CD94/NKG2A and LIR-1 the only known inhibitory receptor present. These cells show consistently high cytotoxic activity against malignant cells, given by high levels of granzymes and activating receptors (Maki et al., 2001). Several phase I and II clinical trials have evaluated NK-92 cells in patients with advanced solid tumors, demonstrating feasibility and a favorable safety profile, although objective clinical responses have been limited (Arai et al., 2008; Klingemann et al., 2016; Tonn et al., 2013).

To address some of the limitations behind poor clinical performance, modern engineered variants have been derived from NK-92 cells and translated into clinical studies. The high-affinity NK (haNK) cells were developed to express a high-affinity CD16 receptor, which enhances ADCC and allows synergistic effects in combination with therapeutic monoclonal antibodies (mAbs). haNK cells also produce intracellular IL-2 to support their survival and cytotoxicity without causing significant systemic IL-2 exposure in the patient (Giles et al., 2019; Jochems et al., 2016). Before infusion, haNK cells are irradiated to prevent proliferation while preserving their short-term antitumor activity and do not lose their cytotoxicity under hypoxic conditions in the TME, in contrast to PB-derived NK cells (Solocinski et al., 2020).

Further genetic engineering has led to the development of more specialized NK cell platforms with expanded therapeutic potential. These include CAR-NK cells, which are engineered to recognize specific tumor antigens, and targeted high-affinity NK (t-haNK) cells, which combine the enhanced ADCC properties of haNK cells with tumor-targeting CAR constructs to improve antitumor specificity and efficacy (Klingemann, 2023; Page et al., 2024). In contrast to CAR-T cells, CAR-NK cells retain their endogenous activating receptors—including NKG2D, NKp30, NKp44, NKp46, DNAM-1, and CD16—allowing simultaneous CAR-dependent and CAR-independent recognition of tumor cells (Balkhi et al., 2025; Lee et al., 2021). Early clinical trials targeting CD19 in B cell malignancies demonstrated high response rates with a striking safety profile, notably lacking the severe CRS and ICANS side effects frequently observed following CAR-T therapies (Brudno and Kochenderfer, 2016; Liu et al., 2020; Tang et al., 2018). Remarkable examples include anti-epithelial cell adhesion molecule (EpCAM), anti-programmed death-ligand 1 (PD-L1) and anti-human epidermal growth factor receptor 2 (HER2) CAR-NK cells (Fabian et al., 2020; Robbins et al., 2020; Sahm et al., 2012; Uherek et al., 2002). A variety of tumor-specific CAR-engineered iPSC-derived NK cells and NK-92 cell “armored” products have been generated to survive the TME and support future clinical development. These CAR-NK cell products include constitutive expression of IL-12, IL-15, IL-18, CCL19, CXCR1, CXCR2, and dominant-negative TGF-β receptors or TGFBR2 knockout to maintain cytotoxic function within TGF-β-rich tumors (Peng et al., 2024; Wang et al., 2024; Zhong and Liu, 2024). It is important to mention that, in CAR-engineered NK cells, endogenous KIR signaling remains functional and is integrated with CAR-mediated activation. Thus, recognition of recipient HLA by inhibitory KIRs can attenuate CAR-NK activity, whereas sufficiently strong CAR signaling may overcome this inhibition, particularly on tumor cells with high target-antigen density. Consequently, KIR–HLA interactions modulate rather than simply determine CAR-NK cytotoxicity and do not constitute an absolute safeguard against recognition of antigen-expressing normal tissues (Zhuang and Long, 2022).

These next-generation NK platforms may be particularly attractive for the treatment of rare and ultra-rare solid tumors because they can be developed as readily available, off-the-shelf cell therapy products, potentially improving accessibility and reducing treatment delays. Unlike conventional CAR-T cells, CAR-NK cells retain endogenous activating and inhibitory receptors that provide additional mechanisms of tumor recognition and regulation. However, this intrinsic signaling does not eliminate the risk of on-target, off-tumor toxicity, particularly when the CAR target antigen is also expressed on healthy tissues. Careful antigen selection and optimization of CAR signaling therefore remain critical to achieving an appropriate therapeutic window.

### 3.2 Cytokine-based NK cell therapy

The administration of exogenous immunostimulatory cytokines aims at enhancing the proliferation, activation, survival, and cytotoxic function of endogenous or adoptively transferred NK cells. With IL-2 the first cytokine used to clinically activate NK cells, administration of IL-12, -15, −18 and −21 rapidly followed for multiple indications and combinations (Floros and Tarhini, 2015). While IL-2 clinical utility was severely limited by its systemic toxicity and preferential expansion of immunosuppressive Tregs, IL-15 has been shown to potently stimulate NK cell activation and survival *in vivo* without significantly expanding Tregs. Several IL-15-based agents–including recombinant IL-15, heterodimeric IL-15/IL-15Rα complexes (e.g., hetIL-15/NIZ985), IL-15 receptor agonists (such as the IL-15 superagonist complex N-803), and membrane-bound IL-15 expressed on engineered NK cells–have demonstrated encouraging preclinical and clinical activity (Gao et al., 2026; Kim et al., 2016; Knudson et al., 2020; Leidner et al., 2023; Wrangle et al., 2018). Notably, pre-activation of NK cells with IL-12, IL-15, and IL-18 generates cytokine-induced memory-like (CIML) NK cells, which exhibit enhanced IFN-γ production, increased cytotoxicity, prolonged persistence, and improved antitumor responses following ACT (Romee et al., 2016).

Building on the improvements of CIML-NK cell programming, next-generation manufacturing platforms have been developed to generate memory cytokine-enriched NK (M-ceNK) cells, designed to enhance therapeutic potential and scalable manufacturing for ACT. M-ceNK cells are generated from leukapheresis products obtained from either healthy donors or cancer patients through *ex vivo* activation with a cytokine cocktail containing N-803, IL-12, and IL-18, yielding a highly purified CD3^neg^CD56^high^ NK cell population. Compared with conventional PB-derived NK cells, M-ceNK cells display a highly activated phenotype characterized by increased expression of the natural cytotoxicity receptors NKp30, NKp44, and NKp46, reduced expression of inhibitory receptors such as KLRG1 and T cell immunoglobulin and ITIM domain (TIGIT), and enhanced production of the effector molecules IFN-γ and granzyme B (Fousek et al., 2026; ImmunityBio, 2021; Singh Nangia et al., 2025). In addition, these cells undergo metabolic reprogramming toward increased glycolytic activity, a feature associated with enhanced activation, proliferative capacity, and sustained effector function. Preclinical studies have demonstrated superior antitumor activity against both hematological and solid tumors, while early-phase clinical studies suggest favorable safety and promising therapeutic efficacy. Cytokine-based approaches are therefore increasingly incorporated into ACT protocols to improve NK cell expansion and durability while enhancing clinical efficacy.

### 3.3 Antibody-based targeting and engagers of NK cells

Several therapeutic mAbs–such as rituximab (anti-CD20), cetuximab (anti-epidermal growth factor receptor (EGFR)) and trastuzumab (anti-HER2) – have demonstrated clinical efficacy that is heavily dependent on NK cell-mediated ADCC. These therapies harness endogenous NK cells by engaging CD16 activating receptors. As a result, tumor cell killing is induced by coating malignant cells with immunoglobulin G (IgG), thereby facilitating NK cell recognition and cytotoxicity (Dixon et al., 2021; Huan et al., 2023). Beyond targeting CD16, other therapeutic mAbs aim to relieve inhibitory signaling by blocking inhibitory receptors on NK cells, including KIRs, NKG2A, LAG-3, TIGIT and CD96 (Andre et al., 2018; Khan et al., 2020). Some of these mAbs are being evaluated in clinical trials as treatment for advanced solid tumors (Galot et al., 2021; Tinker et al., 2019; Vey et al., 2018), with clinical success yet to be proven. Engineered multispecific antibodies have been developed to redirect NK cells to tumor cells, in an approach that highly resembles the bispecific T cell engager (BiTE) format designed for T cells. Bispecific killer engagers (BiKEs) simultaneously bind CD16 on NK cells and tumor-associated antigens (TAAs), aiming to bring NK cells and target cells into proximity, inducing the formation of cytolytic synapses that ultimately result in tumor cell death (Li et al., 2020). Tri-specific killer engagers (TriKEs) have also been investigated, featuring a trivalent design incorporating a third single-chain variable fragment (scFv) targeting another TAA or cell-stimulating cytokines like IL-15 to enhance NK cell proliferation and persistence while promoting cytotoxicity (Felices et al., 2016; Hodgins et al., 2019). For instance, the CD16×EpCAM BiKE was developed to redirect NK cell ADCC against EpCAM-positive carcinoma cells and further engineered by adding an IL-15 cross-linker to produce the 1615EpCAM TriKE, which improved NK cell expansion, activation, survival, and killing of EpCAM-positive tumor cells (Schmohl et al., 2016; Vallera et al., 2013). These engagers have shown encouraging preclinical antitumor activity in multiple solid tumors, and several others have entered early-phase clinical trials. The camelid anti-CD16×IL-15×B7-H3 TriKE, also referred to as GTB-5550, is currently under investigation in a first-in-human phase 1a/1b trial for the treatment of advanced solid tumors (NCT07541573). Similarly, early studies with the EGFR × CD16A bispecific engager (also known as AFM24) reported disease stabilization and some partial responses, particularly in combination with checkpoint inhibitors or ACT, against advanced/metastatic EGFR-expressing tumors (El-Khoueiry et al., 2025). Despite the favorable safety profile with limited cytokine release, response rates remain modest, and the study has not yet demonstrated the kind of efficacy comparable with approved T cell engagers. Thus far, however, no NK engager has yet demonstrated unequivocal clinical success or received regulatory approval for a solid tumor indication.

## 4 Clinical development of adoptive NK cell therapies for solid tumors

The current clinical landscape illustrates several distinct engineering strategies that seek to improve antigen recognition, persistence, or resistance to the TME. Clinical translation of adoptive NK cell therapies for solid tumors has accelerated substantially in recent years, with multiple engineering strategies entering clinical evaluation (Figure 2). Among these, PD-L1 t-haNK cells represent the most clinically advanced platform. PD-L1 t-haNK cells are derived from the irradiated NK-92 cell line and are genetically engineered to express a high-affinity, non-cleavable CD16 Fc receptor, endogenous IL-2 to support autonomous survival, and a CAR directed against PD-L1. Because PD-L1 expression is induced by inflammatory cytokines such as IFN-γ and is frequently upregulated in response to immune checkpoint blockade, chemotherapy, or radiotherapy, PD-L1 t-haNK cells are particularly attractive as combination partners capable of targeting adaptive immune resistance while simultaneously exploiting ADCC (Fabian et al., 2020). The first clinical evaluation of this platform was conducted in the first-in-human phase 1 QUILT-3.064 trial (NCT04050709), which enrolled patients with locally advanced or metastatic solid tumors that had progressed after standard therapies. The primary objectives were to establish safety, tolerability, feasibility, and recommended phase 2 dose (RP2D) of repeated intravenous PD-L1 t-haNK infusions. This study demonstrated that repeated administration of PD-L1-directed CAR-NK product was feasible, with an acceptable safety profile and no evidence of the severe toxicities commonly associated with CAR-T cell therapies. Preliminary evidence of disease stabilization in some heavily pretreated patients further supported continued clinical development, although objective efficacy was not the primary endpoint of this dose-escalation study. These findings established the clinical foundation for subsequent disease-specific phase 2 trials (Seery et al., 2020). The phase 2 QUILT-88 trial (NCT04390399) is investigating PD-L1 t-haNK cells in locally advanced or metastatic pancreatic ductal adenocarcinoma (PDAC). This study incorporates the PD-L1 t-haNK cells into a multimodal immunotherapy regimen that combines standard chemotherapy with the IL-15 superagonist N-803, low-dose cyclophosphamide, stereotactic body radiation therapy, and aldoxorubicin. The rationale for this complex combination is to remodel the TME by enhancing antigen release, promoting NK cell activation and expansion through IL-15 signaling, reducing immunosuppressive Tregs, and increasing PD-L1 expression following chemo-radiotherapy, thereby creating a more favorable environment for PD-L1-targeted NK cell cytotoxicity. The randomized, multi-cohort design evaluates this strategy across first-line maintenance, second-line, and later-line treatment settings, highlighting efforts to integrate NK cell therapy into multiple stages of pancreatic cancer management.

**FIGURE 2:**
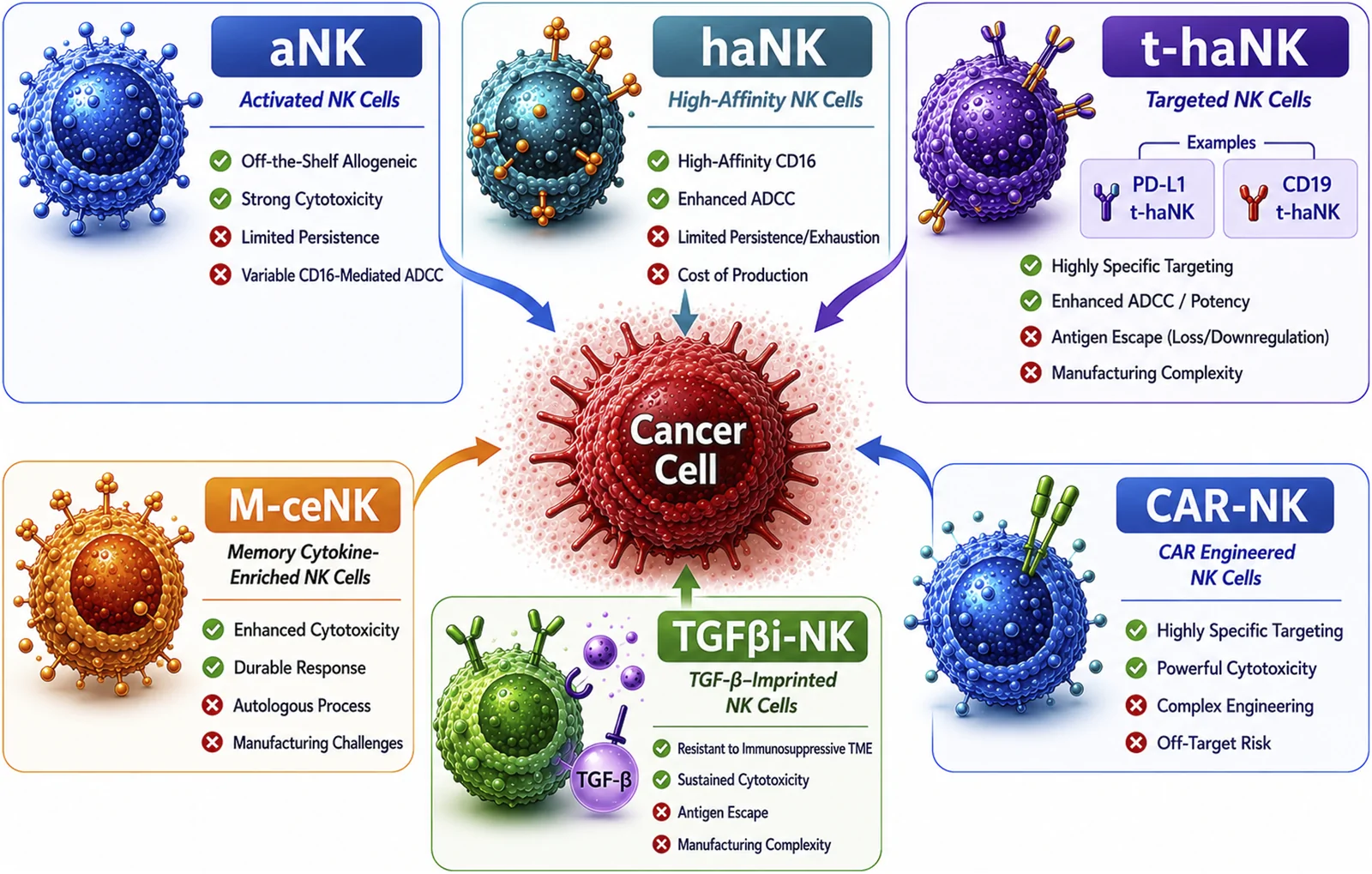
Engineered NK cell platforms for enhanced antitumor activity. Schematic overview of representative engineered NK cell platforms designed to improve the therapeutic efficacy of NK cell-based therapies. Activated NK (aNK) cells, high-affinity NK (haNK) cells, targeted high-affinity NK (t-haNK) cells, memory cytokine-enriched NK (M-ceNK) cells, TGFβ-imprinted NK (TGFβi-NK) cells, and chimeric antigen receptor-engineered NK (CAR-NK) cells employ complementary strategies to enhance tumor recognition, cytotoxicity, persistence, and resistance to the immunosuppressive tumor microenvironment (TME). Each platform incorporates distinct biological modifications and manufacturing approaches, highlighting the expanding repertoire of next-generation NK cell products for cancer treatment. ADCC, antibody-dependent cellular cytotoxicity; TGFβ, transforming growth factor-beta.

The clinical development of PD-L1 t-haNK cells has also expanded into head and neck squamous cell carcinoma (HNSCC), a disease in which PD-L1 expression is common and immune checkpoint inhibitors have become part of standard treatment but provide durable responses in only a minority of patients. The ongoing phase 2 trial in resectable HNSCC (NCT06161545) investigates PD-L1 t-haNK cells in the perioperative (neoadjuvant and/or adjuvant) setting, testing whether the therapeutic combination can reduce tumor size or alter its biology before standard surgery, when TAAs remain available to stimulate systemic immune responses and potentially eradicate micrometastatic disease. Within this immunologically favorable window, investigators aim to improve pathological responses and reduce postoperative recurrence. Complementing this strategy, another phase 2 study (NCT06239220) evaluates PD-L1 t-haNK cells in recurrent or metastatic HNSCC, where therapeutic options remain limited following progression on platinum-based chemotherapy and immune checkpoint inhibitors. In this advanced disease setting, PD-L1-targeted NK cell therapy is expected to overcome several mechanisms of resistance to programmed death-1 (PD-1)/PD-L1 blockade and extend the benefits of immunotherapy beyond the subset of patients who respond to checkpoint inhibition alone.

The broad therapeutic potential of PD-L1-directed NK cell therapy is further illustrated by the ongoing multicenter phase 2b QUILT-3.055 study (NCT03228667), which is evaluating PD-L1 t-haNK cells across a diverse range of PD-L1-expressing malignancies, including non-small cell lung cancer, small cell lung cancer, HNSCC, HCC, renal cell carcinoma, Merkel cell carcinoma, colorectal cancer, urothelial carcinoma, melanoma, gastric cancer, and cervical cancer. This basket-style design therefore aims to establish whether PD-L1 expression can serve as a pan-tumor therapeutic target for adoptive NK cell therapy. Moreover, because PD-L1 expression is frequently increased following chemotherapy, radiotherapy, or checkpoint blockade, this strategy may be particularly valuable in combination regimens that intentionally enhance target antigen expression while simultaneously sensitizing tumors to NK cell-mediated cytotoxicity. Collectively, these ongoing trials underscore the versatility of PD-L1 t-haNK cells and position them as one of the leading off-the-shelf CAR-NK platforms advancing through clinical development. Although PD-L1 t-haNK cells currently represent the most clinically advanced engineered NK platform, several additional NK cell technologies have recently entered clinical evaluation.

As CAR-T cell therapies advanced through development and early clinical trial results emerged, it became evident that their high manufacturing costs, together with the risks of CNS and ICANS, provided strong impetus to explore NK cells–and particularly the NK-92 cell line–as an alternative platform for CAR-engineered cytotoxic immune cell therapies (Pfefferle and Huntington, 2020). The PRISM-NK clinical trial (NCT07510828) is an active phase 1/2 trial investigating precision-matched, allogeneic CAR-NK cells, currently recruiting patients with advanced, treatment-refractory solid tumors. Here, participants are assigned to receive either a single-target or dual-target CAR-NK cell product based on tumor antigen expression. The phase 1 portion of the study employs a conventional 3 + 3 dose-escalation design within each treatment arm to determine the RP2D. In phase 2, target-specific expansion cohorts are treated at the RP2D to further evaluate safety and obtain preliminary evidence of antitumor efficacy. A phase 2 trial of PD-L1 CAR-NK cells (NCT04847466) has recently concluded in patients with recurrent or metastatic gastroesophageal junction (GEJ) cancer or advanced HNSCC, leveraging the frequent overexpression of PD-L1 in these malignancies. The trial has been designed to test the effectiveness of irradiated PD-L1 CAR-NK cells in combination with pembrolizumab and N-803, assessing the safety and tolerability of the combination therapy as well as progression-free survival and duration of response in enrolled patients. Similarly, trophoblast cell surface antigen 2 (TROP2)-targeted, engineered IL15-transduced CB-derived CAR-NK cells, which recognize a TAA commonly expressed in epithelial cancers, are being evaluated in a phase 1 dose escalation and expansion trial involving advanced non-small cell lung cancer and HER2-negative or HER2-low breast cancers, including triple-negative breast cancer (NCT06066424), to determine the safety, tolerability, optimal cell dose, maximum tolerated dose, and RP2D of TROP2-CAR-NK cells in participants with solid tumors characterized by high TROP2 expression. Additionally, objective response rates in patients treated with TROP2-CAR-NK cells are being evaluated in a phase 1/2 study for platinum-resistant high-grade ovarian cancer, mesonephric-like adenocarcinoma, and pancreatic cancer (NCT05922930).

In an effort to enhance NK cell persistence and activation through cytokine engineering, CIML-NK cells were originally developed from the observation that brief preactivation with IL-12, IL-15, and IL-18 induces a durable “memory-like” state marked by enhanced secondary cytokine production, cytotoxicity, and responsiveness to low-dose IL-2. Preclinical work also showed that these cells can persist better and expand more effectively after adoptive transfer, providing the biologic foundation for several of the early-phase trials discussed below. In QUILT-3.076 (NCT04898543), an open-label phase 1 study, autologous M-ceNK cells are being evaluated in patients with locally advanced or metastatic solid tumors together with the subcutaneous IL-15 superagonist N-803. The follow-on phase 2 study in platinum-resistant high-grade ovarian cancer (NCT06710288) pushes the same platform into a more disease-specific setting in which autologous M-ceNK cells are combined with gemcitabine and N-803 in a single-arm trial. Likewise, the sIL15_TRACK NK cells trial (NCT05334329) represents a different engineering solution to the persistence problem. In this phase 1 study for non-small cell lung cancer that has already progressed on PD-1/PD-L1 therapy, umbilical CB-derived NK cells are engineered to express soluble IL-15 (sIL-15) and PD-L1, and they are administered with or without atezolizumab (anti-PD-L1) after lymphodepleting fludarabine/cyclophosphamide. The trial’s primary aims are safety, optimal biological dose, and peripheral blood persistence, while correlative analyses examine cytokines, immune cell activation, and tumor trafficking. Early clinical data published in an interim report of six patients confirm that the cells were detectable shortly after infusion, were found in a tumor biopsy after treatment, retained cytolytic activity, and were associated with disease stabilization in half of the cohort (Villalona-Calero et al., 2025). These findings make this platform especially interesting as a proof-of-concept for combining cytokine engineering with checkpoint blockade in an immune-refractory solid tumor.

The CIML-NK ovarian cancer study (NCT06321484) extends the same memory-like biology into the peritoneal cavity, which is a logical route for recurrent ovarian cancer. This single-site phase 1b study uses leukapheresis, lymphodepleting chemotherapy, intraperitoneal CIML NK-cell infusion, and low-dose IL-2 support, with a 3 + 3 dose-escalation framework and long-term follow-up. The intraperitoneal approach is notable because ovarian cancer often remains anatomically confined to the peritoneal space even when it is biologically advanced, so local delivery may improve NK cell exposure at the tumor surface while minimizing reliance on systemic trafficking alone. In parallel, ML-NK cells are being assessed in a phase 1 trial for advanced or metastatic melanoma (NCT05629546) together with nivolumab and relatlimab in patients with advanced or metastatic melanoma that has progressed after checkpoint inhibitors, extending this strategy to immune-responsive solid tumors.

To overcome immunosuppressive signaling within the tumor microenvironment, TGFβ-imprinted (TGFBi) NK cells have been engineered to resist TGFβ-mediated inhibition. Current clinical evaluation includes a phase 1b/2 trial in metastatic HER2-negative breast cancer (NCT06026657), a phase 1 study in cutaneous squamous cell carcinoma and basal cell carcinoma (NCT07144384), and a phase 1/2 trial for patients with stage IV melanoma metastatic to the brain (NCT05588453). These studies aim to determine whether resistance to TGF-β signaling can enhance NK cell persistence and function in tumors that generally present highly immunosuppressive TME (Table 1). It is crucial to consider that, despite the important advance, the TGF-β signaling pathway represents only one component of a highly redundant suppressive network characterized by metabolically unfavorable conditions and might not be sufficient to achieve durable tumor control.

**TABLE 1:** Recent clinical trials of NK cell-based therapies in solid tumors.

NK therapy platform	Trial identifier	Primary indication	Phase	Status
PD-L1 t-haNK	NCT04050709	Locally advanced or metastatic solid cancers	Phase 1	Completed (2026)
	NCT04390399	Locally advanced or metastatic pancreatic cancer	Phase 2	Active, not recruiting
	NCT06161545	Resectable HNSCC	Phase 2	Recruiting
	NCT06239220	R/M HNSCC	Phase 2	Recruiting
	NCT03228667	NSCLC, SCLC, HNSCC, HCC, RCC, MCC, CRC, urothelial carcinoma, melanoma, gastric cancer, cervical cancer	Phase 2b	Active, not recruiting
CAR-NK	NCT07510828	Advanced, treatment-refractory solid tumors	Phase ½	Recruiting
PD-L1 CAR-NK	NCT04847466	R/​M GEJ cancer or advanced HNSCC	Phase 2	Completed (2026)
TROP2- CAR-NK	NCT06066424	Advanced solid tumors (NSCLC, HER2-negative/low breast cancer, TNBC)	Phase 1	Recruiting
	NCT05922930	Platinum-resistant HGOC	Phase ½	Recruiting
M-ceNK	NCT04898543	Locally advanced or metastatic solid tumors	Phase 1	Active, not recruiting
	NCT06710288	Platinum-resistant HGOC	Phase 2	Recruiting
sIL15_TRACK NK	NCT05334329	Advanced or R/M NSCLC	Phase 1	Active, not recruiting
CIML-NK	NCT06321484	Recurrent HGOC	Phase 1b	Recruiting
	NCT06318871	RCC and urothelial carcinoma	Early phase 1	Active, not recruiting
TGFBi NK	NCT06026657	Metastatic, HER2-negative breast cancer	Phase 1b/2	Recruiting
	NCT07144384	Cutaneous SCC or BCC	Phase 1	Recruiting
	NCT05588453	Stage IV melanoma metastatic to the brain	Phase ½	Recruiting
ML NK	NCT05629546	Advanced or metastatic melanoma	Phase 1	Recruiting

Data compiled from ClinicalTrials.gov, last updated in July 2026. Searches included terms related to natural killer (NK) cell therapy and solid tumors, including “natural killer cells,” “NK cells therapies,” “CAR-NK,” and related engineered NK cell platforms. Eligible studies were interventional clinical trials evaluating adoptive NK cell-based therapies in patients with solid malignancies. Studies restricted to hematologic malignancies, observational studies, and studies not involving therapeutic NK cell administration were excluded. Trial phase, indication, recruitment status, and ClinicalTrials.gov identifier were verified against the corresponding ClinicalTrials.gov record at the time of the final search. BCC, basal cell carcinoma; CAR, chimeric antigen receptor; CIML, cytokine-induced memory-like; CRC, colorectal cell carcinoma; GEJ, gastroesophageal junction; HCC, hepatocellular carcinoma; HER2, human epidermal growth factor receptor 2; HGOC, high-grade ovarian cancer; HNSCC, head and neck squamous cell carcinoma; MCC, Merkel cell carcinoma; M-ceNK, Memory cytokine-enriched natural killer; ML, memory-like; NSCLC, non-small cell lung cancer; PD-L1, programmed death ligand 1; RCC, renal cell carcinoma; R/M, recurrent or Metastatic; SCC, squamous cell carcinoma; SCLC, small cell lung carcinoma; sIL15, soluble interleukin 15; TGFβi, transforming growth factor beta imprinted; t-haNK, targeting high-affinity natural killer; TNBC, triple negative breast cancer; TRACK, tumor-reactive and cytokine-activated killer; TROP2, trophoblast cell surface antigen 2.

Collectively, these studies suggest that the next-generation of NK cell therapies is moving beyond antigen recognition. Current and future development encompasses multifunctional engineering that combines enhanced tumor trafficking, resistance to dominant suppressive pathways, improved persistence via cytokine support, checkpoint disruption and metabolic reprogramming.

## 5 The challenge of treating rare and ultra-rare solid tumors

### 5.1 Clinical rationale for adoptive NK cell therapy

Rare and ultra-rare tumors pose significant challenges for clinical development due to their low incidence, biological heterogeneity, and limited clinical evidence (Blay et al., 2024; Casali and Trama, 2020). The National Cancer Institute (NCI) currently defines a “rare cancer” as one with an annual incidence of fewer than 15 cases per 100,000 individuals, whereas the more recently introduced term “ultra-rare cancer” refers to malignancies with an annual incidence of fewer than 1 case per 1,000,000 individuals. Rather than representing a single disease category, rare cancers encompass hundreds of biologically distinct malignancies with diverse molecular alterations and tumor immune microenvironments. Consequently, therapeutic approaches frequently require tumor-specific rather than broadly applicable design and optimization (Boyd et al., 2016). Rare and ultra-rare cancers often include pediatric malignancies, sarcomas, and distinct central nervous system (CNS) tumors (Table 2), for which the implementation of ACT presents several issues.

**TABLE 2:** Classification of rare and ultra-rare solid tumors.

Tumor type	Category	Disease incidence (per 1,000,000)	Estimated U.S. cases/year
Adrenocortical carcinoma	Ultra-rare endocrine cancer	1–2	350–700
Ampullary carcinoma	Rare GI cancer	5–10	2,000–3,000
Chordoma	Ultra-rare bone tumor	0.8–1	300
Fibrolamellar HCC and HEH	Ultra-rare liver cancers	0.2–1	200–300
Glioblastoma (GBM)	Rare brain cancer	30–40	12,000–15,000
Merkel cell carcinoma	Rare skin cancer	8–10	2,500–3,500
Neuroendocrine/Chromaffin Tumors	Rare tumors	2–8	700–2,500
NUT carcinoma	Ultra-rare squamous cell cancer	0.1–0.2	<100
Salivary gland cancers	Rare cancers	3–5	2,000–3,000
Small bowel adenocarcinoma	Rare GI cancer	20–30	7,000–10,000
Soft tissue & bone sarcomas	Rare solid tumors	150–200	50,000–65,000
Thymic malignancies	Rare thoracic cancers	4–5	1,500
Uveal melanoma	Rare ocular cancer	5–7	1,500–2,000

Data compiled from SEER, Incidence data (NCI) and (DeSantis et al., 2017). HCC, hepatocellular carcinoma; HEH, hepatic epithelioid hemangioendothelioma; NUT, nuclear protein in testis; GI, gastrointestinal.

One of the defining characteristics is the extremely small patient population, creating substantial economic and logistical barriers to the development of personalized therapies. There is historically less commercial incentive to develop patient-specific, labor-intensive, and costly therapies such as autologous CAR-T cells, for example. Rare and ultra-rare tumors frequently lack well-characterized tumor-specific neoantigens while they often express antigens that are also present on normal tissues, thus creating an additional obstacle. This overlap creates a significant risk of “on-target, off-tumor” toxicity, in which engineered immune cells damage healthy organs expressing the same antigen. Target discovery is further complicated by the limited availability of patient-derived biospecimens, representative preclinical models, and comprehensive molecular datasets, restricting the identification and validation of actionable therapeutic targets. Furthermore, pediatric and medically fragile patients are at increased risk of experiencing severe treatment-related toxicities associated with conventional T cell therapies (Brudno and Kochenderfer, 2024; Shalabi et al., 2021). Many rare solid tumors possess highly immunosuppressive microenvironments characterized by dense stromal architecture, suppressive myeloid populations, inhibitory cytokines, and poor immune cell infiltration, all of which limit the persistence and cytotoxic activity of adoptively transferred lymphocytes.

Engineered NK cells offer a compelling paradigm shift for these orphan diseases, mostly through allogeneic off-the-shelf manufacturing and distinct safety profiles. Clinical studies have shown that CB-derived CAR-NK cells did not elicit adverse effects in patients, namely CRS, ICANS or GVHD (NCT03056339) (Liu et al., 2020). Pediatric patients with relapsed or refractory solid tumors also exhibited a favorable safety profile following treatment with CB-derived NK cells, with no reported cases of CRS or neurotoxicity, and donor NK cells remaining detectable in peripheral blood for up to 9 days after infusion (NCT03420963). Unlike T cell-based therapies, which depend almost exclusively on antigen recognition, the antitumor activity of NK cell-based platforms relies on an array of activating and inhibitory receptors that recognize a broad range of stress signals, loss of MHC molecules and other indicators of malignant transformation. The result of this innate recognition is an additional mechanism for identifying abnormal cells beyond antigen targeting (Zhong and Liu, 2024).

### 5.2 Current landscape of NK cell therapies in rare and ultra-rare solid tumors

The clinical development of NK cell-based therapies for rare and ultra-rare solid tumors has expanded considerably in recent years. NK cell therapies may be particularly well suited for rare malignancies because they can be rapidly retargeted to diverse TAAs across histologies, combined with monoclonal antibodies or local treatment modalities, and manufactured as allogeneic off-the-shelf products (Lupo and Matosevic, 2019; Oh et al., 2019). The ongoing clinical progress has been driven by a substantial body of preclinical work demonstrating that NK cells can overcome the intrinsic resistance of these malignancies to conventional therapies. Multiple early-phase clinical trials evaluating diverse NK cell platforms across a range of rare and ultra-rare solid tumors are currently in the recruitment phase, reflecting the rapid expansion of the field despite its relatively early stage of development (Table 3). However, achieving durable clinical responses will require overcoming key barriers, including limited tumor trafficking and infiltration, insufficient *in vivo* persistence, and the immunosuppressive TME (Figure 3) (Dagher and Posey, 2023; Portillo et al., 2023).

**TABLE 3:** Recent clinical trials of NK cell-based therapies in rare and ultra-rare solid tumors.

NK therapy platform	Trial identifier	Primary indication	Phase	Status
PD-L1 t-haNK	NCT06061809	Recurrent or progressive GBM	Phase 2	Recruiting
NK-92/5.28.z	NCT03383978	Recurrent HER2-positive GBM	Phase 1	Recruiting
TGFβi NK	NCT04211675	R/R Neuroblastoma	Phase 1	Recruiting
	NCT04254419	Recurrent, progressive, or refractory CNS tumors	Phase 1	Recruiting
	NCT05634369	R/R sarcomas	Phase 2	Recruiting
[CB]-NK-TGF-betaR2-/NR3C1	NCT04991870	Recurrent, grade 4 astrocytoma	Phase 1	Recruiting
NY-ESO-1 TCR/IL-15 NK	NCT06083883	Advanced synovial sarcoma and myxoid/round cell liposarcoma	Phase 1/1b	Recruiting
CAR.70/IL15-transduced CB-NK	NCT05703854	Mesothelioma Osteosarcoma	Phase 1/2	Recruiting
Allogeneic NK	NCT06687681 NCT06450041 NCT05887882	Others	Phase 1/2	Recruiting Recruiting Suspended

Data compiled from ClinicalTrials.gov, last updated in July 2026. Searches included terms related to natural killer (NK) cell therapy and solid tumors, including “natural killer cells,” “NK cells therapies,” “CAR-NK,” and related engineered NK cell platforms. Eligible studies were interventional clinical trials evaluating adoptive NK cell-based therapies in patients enrolled in trials involving rare and ultra-rare solid malignancies. Studies restricted to hematologic malignancies, observational studies, and studies not involving therapeutic NK cell administration were excluded. Trial phase, indication, recruitment status, and ClinicalTrials.gov identifier were verified against the corresponding ClinicalTrials.gov record at the time of the final search. CAR, chimeric antigen receptor; CB, cord blood-derived; CNS, central nervous system; GBM, glioblastoma; HER2, human epidermal growth factor receptor 2; IL-15, interleukin 15; NR3C1, nuclear receptor subfamily 3 group C member 1; NY-ESO-1, New York esophageal squamous cell carcinoma-1 antigen; PD-L1, programmed death ligand 1; R/R, relapsed and refractory; TGFβi, transforming growth factor beta imprinted; TGF-betaR2, transforming growth factor beta receptor 2; TCR, T cell receptor; t-haNK, targeting high-affinity natural killer.

**FIGURE 3:**
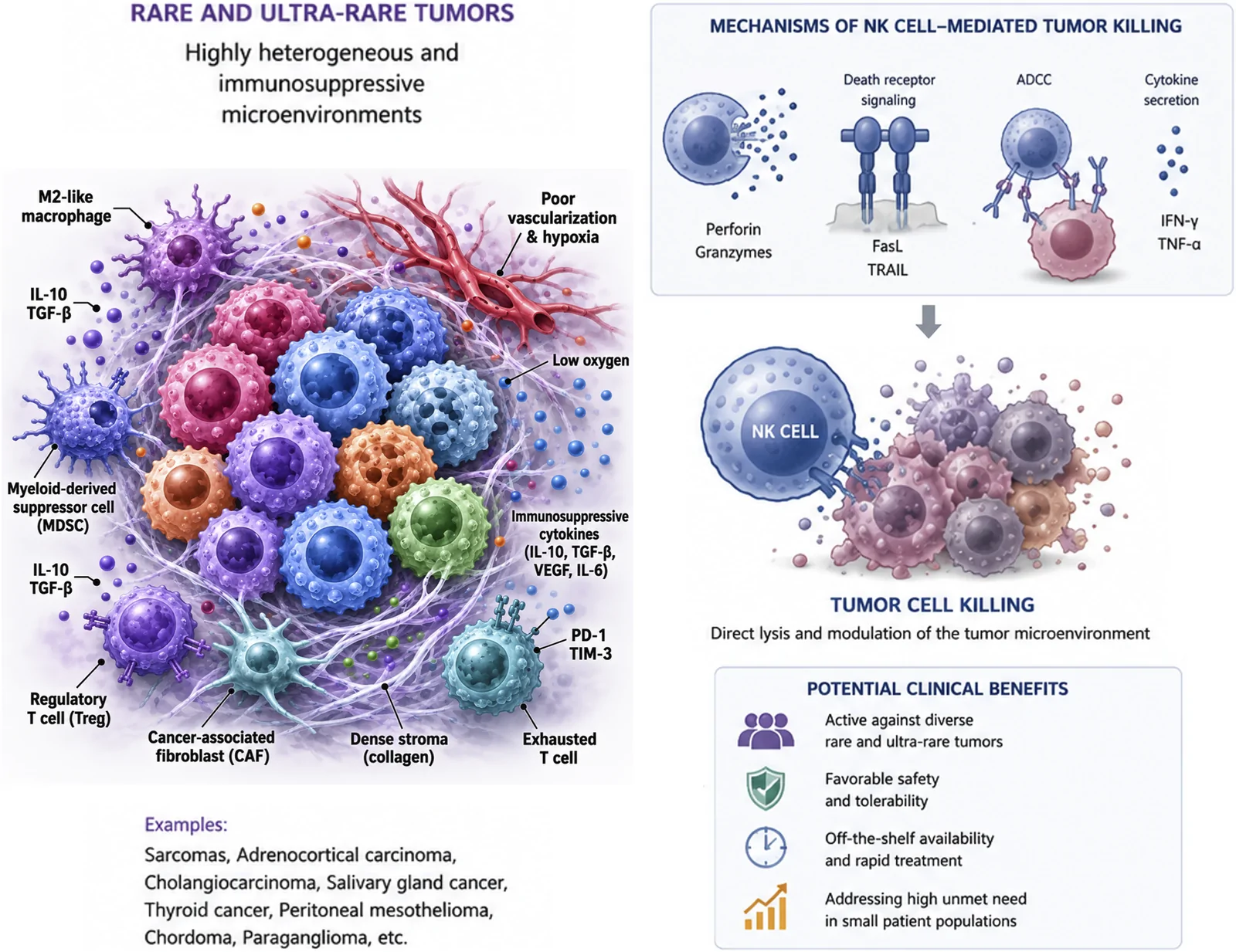
Overcoming the immunosuppressive TME in rare and ultra-rare solid cancers with adoptive NK cell therapy. Rare and ultra-rare tumors frequently establish profoundly immunosuppressive tumor microenvironments (TMEs) characterized by stromal desmoplasia, hypoxia, suppressive myeloid and regulatory immune populations, and inhibitory cytokines that limit effective immune responses. Adoptively transferred NK cells retain the capacity to overcome these barriers through multiple mechanisms, including perforin/granzyme-mediated cytotoxicity, death receptor signaling (FasL/TRAIL), antibody-dependent cellular cytotoxicity (ADCC), and secretion of pro-inflammatory cytokines such as IFN-γ and TNF-α. Together, these mechanisms promote direct tumor cell lysis and remodeling of the TME, supporting the development of off-the-shelf NK cell therapies as a promising treatment strategy for rare and ultra-rare malignancies with high unmet clinical need. IFN-γ, interferon gamma; TNF-α, tumor necrosis factor; TRAIL, tumor necrosis factor–related apoptosis-inducing ligand.

Glioblastoma (GBM) has emerged as one of the leading models for clinical evaluation of engineered NK cell therapies. Current trials evaluating PD-L1 t-haNK cells and intracranial CAR-NK cells targeting HER2 are the result of years of preclinical optimization aimed at improving tumor infiltration and persistence in patients with recurrent or progressive GBM. The phase 2 QUILT-3.083 trial (NCT06061809) builds upon extensive preclinical evidence demonstrating that PD-L1 expression is frequently induced in GBM by inflammatory cytokines, radiotherapy, and temozolomide, contributing to adaptive immune resistance and suppression of antitumor T cell responses (Wang et al., 2019). The trial incorporates PD-L1 t-haNK cells into a multimodal treatment regimen that includes N-803 and additional immune-modulating agents designed to enhance NK cell activation, persistence, and trafficking within the highly immunosuppressive GBM microenvironment. A complementary strategy focuses on HER2-directed CAR-NK therapy. The ongoing phase 1 CAR2BRAIN trial (NCT03383978) is evaluating NK-92/5.28.z cells, an irradiated NK-92 cell line genetically engineered to express a second-generation CAR recognizing HER2, in patients with recurrent HER2-positive GBM. HER2 is expressed in a substantial subset of GBMs while exhibiting limited expression in normal brain tissue, making it an attractive therapeutic target despite its heterogeneous distribution within tumors. Importantly, this study departs from conventional intravenous administration by delivering CAR-NK cells directly into the resection cavity through an implanted intracranial catheter. This local delivery strategy was developed to circumvent one of the major limitations of systemic cellular immunotherapy for brain tumors–the restricted migration of effector cells across the blood brain barrier–and to maximize NK cell exposure within the residual tumor bed, where recurrence most commonly occurs.

Similarly, engineered TGFβi NK cells are under investigation across multiple pediatric and adult malignancies, including relapsed/refractory neuroblastoma, recurrent or refractory CNS tumors, and relapsed/refractory sarcomas (NCT04211675, NCT04254419, NCT05634369). Translational progress has also been observed in malignant meningioma, where Giles et al. demonstrated that the anti-PD-L1 mAb avelumab effectively directs NK cell-mediated ADCC against PD-L1-expressing aggressive tumors. Furthermore, the study highlighted how engineered haNK cells consistently outperformed peripheral blood NK cells in meningioma ADCC, providing a compelling rationale for combining checkpoint blockade with engineered NK cell therapy in CNS tumors (Giles et al., 2019). Next-generation NK cell platforms are extending beyond conventional CAR engineering by incorporating genetic modifications designed to overcome hostile TMEs. For instance, a phase 1 study in patients with recurrent grade 4 astrocytoma is evaluating CB- derived NK cells engineered with a dominant-negative TGFβR2 together with NR3C1 modification (NCT04991870). This dual-engineering strategy addresses two major mechanisms of immune suppression within gliomas. The dominant-negative TGFβR2 blocks inhibitory TGF-β signaling, thereby preserving NK cell activation, cytotoxicity, and cytokine production in the tumor. In parallel, NR3C1 modification reduces glucocorticoid-mediated suppression of NK cell function, an important consideration because many patients with high-grade glioma require prolonged corticosteroid therapy for cerebral edema. Collectively, these modifications are intended to maintain NK cell functionality within an otherwise profoundly suppressive CNS tumor microenvironment.

A similar bench-to-bedside progression is occurring in chordoma, a rare bone malignancy characterized by resistance to chemotherapy and radiotherapy. Initial studies demonstrated that PD-L1 expression on chordoma cells can be upregulated by IFN-γ, leading to a significantly increased sensitivity to ADCC when the anti-PD-L1 mAb avelumab and NK cells were present (Fujii et al., 2016). Subsequent work expanded this strategy by targeting EGFR with the mAb cetuximab in combination with haNK cells, resulting in substantial cancer cell lysis observed in ADCC and highlighting a potential target for therapeutic intervention in this rare disease (Fujii et al., 2018). Lopez et al. further characterized the chordoma cancer stem cell compartment, responsible for treatment resistance and disease recurrence. The identification of preferential expression of PD-L1, together with NK-activating ligands including B7-H6, MICA/B, and ULBP1 on chordoma cells has suggested that NK cell therapies may simultaneously eliminate bulk tumor cells and therapy-resistant stem-like populations, providing an important mechanistic explanation for durable responses that current clinical studies seek to achieve (Lopez et al., 2024). Comparable translational principles are emerging in other ultra-rare head and neck malignancies, where some of the first preclinical investigation is currently guiding the development of combinatory immunotherapy approaches. In sinonasal undifferentiated carcinoma, anti-PD-L1 antibodies markedly enhanced NK cell-mediated ADCC, while the IL-15 superagonist N-803 further amplified NK cell activation and IFN-γ production, resulting in increased PD-L1 expression on tumor cells and a positive feedback loop that significantly improved tumor killing. Combination treatment with anti-PD-L1 blockade and IL-15 stimulation produced substantially greater cytotoxicity than either approach alone, while analyses of the TME identified endogenous NK cell infiltration and favorable NK-tumor interactions in patients without recurrence (Hoke et al., 2024). Recently, ephrin type-A receptor-2 (EphA2)-targeted CAR-NK cells have demonstrated efficacy against preclinical models of EphA2 antigen-positive HNSCC (Jacobs et al., 2023), and were further applied to the treatment of pediatric sarcomas. The study led by Lam et al. investigated a range of EphA2-targeting CAR designs to improve NK cell cytotoxicity against pediatric sarcoma in *in vitro* and *in vivo* models and further explored chemical mRNA modifications as a strategy to improve the stability and persistence of CAR expression. These findings contribute to the enhancement of therapeutic efficacy and durability of EphA2-CAR-NK cell therapies as well as their advancement toward clinical application (Lam et al., 2025).

Increasingly sophisticated multifunctional NK constructs allow enhanced NK cell activation, improved ADCC, and superior persistence. The ongoing phase I trial NCT05703854 is assessing preliminary antitumor activity, optimal cell dose, safety and tolerability of CAR.70/IL15-transduced CB-NK cells following lymphodepleting chemotherapy in patients with advanced renal cell carcinoma, mesothelioma, or osteosarcoma. These allogeneic NK cells are engineered to target CD70, a ligand frequently overexpressed across several solid tumors but minimally expressed in most normal tissues, providing a favorable therapeutic window. In addition to CAR-mediated antigen recognition, constitutive IL-15 expression is incorporated to improve NK cell persistence and sustained cytotoxic activity after infusion. Lymphodepleting fludarabine and cyclophosphamide are administered before cell infusion to reduce host immune rejection and create a favorable cytokine milieu for NK cell expansion. At the same time, adoptive NK cell therapy is being adapted to intracellular tumor antigens through TCR-based engineering. The phase I trial NCT06083883 is investigating NY-ESO-1 TCR/IL-15-expressing NK cells in patients with advanced synovial sarcoma and myxoid/round cell liposarcoma. Unlike conventional CAR constructs that recognize cell-surface antigens, engineered TCRs enable recognition of intracellular cancer-testis antigens on MHC molecules, substantially expanding the range of targetable tumor antigens. Coexpression of IL-15 is designed to enhance NK cell survival and persistence after infusion without requiring exogenous cytokine administration, thereby addressing one of the major limitations of adoptively transferred NK cells.

Accumulating preclinical evidence and early-phase clinical studies demonstrate that NK cell-based therapies, particularly CAR-NK platforms, represent a versatile and adaptable immunotherapeutic strategy. By enabling tumor-specific targeting through customizable antigen recognition, these approaches have the potential to address the unmet therapeutic needs of rare and ultra-rare malignancies for which conventional drug development is often difficult, thereby expanding treatment opportunities for patients with otherwise limited options.

## 6 Limitations and future directions

Despite remarkable progress in hematologic malignancies, clinical translation of adoptive NK cell therapies to solid tumors has been considerably more challenging. The multiple anatomical, molecular, and immunological barriers presented by solid tumors collectively limit NK cell trafficking and infiltration into tumor tissue. NK cells expanded *ex vivo* frequently lack receptors to tumor-secreted chemokines, resulting in poor homing following systemic administration. Even when NK cells successfully infiltrate tumors, immunosuppressive soluble mediators and metabolic constraints of the TME rapidly impair their function. Here, chronic antigen exposure and persistent activation contribute to NK cell exhaustion and reduced cytotoxic potential (Deng et al., 2025; Kirtane et al., 2021). In translational research, solid tumor heterogeneity presents another major challenge. While hematological malignancies often express relatively uniform lineage markers, solid tumors display substantial spatial and temporal variability in TAA expression. Downregulation or shedding of stress ligands, mutations in antigen presentation pathways, and selective outgrowth of resistant tumor cell clones allow immune escape following NK cell therapy. This problem is particularly pronounced in highly heterogeneous malignancies such as glioblastoma, osteosarcoma, soft tissue sarcomas, and metastatic melanoma, where no single antigen is universally expressed. The difficulty is further compounded in the poorly characterized immune biology of rare and ultra-rare tumors and the scarcity of validated NK-specific targets (Tao et al., 2025).

The next-generation of NK cell therapies is specifically addressing these limitations, and new designs are emerging to overcome the barriers imposed by solid tumors. Advances in gene-editing technologies are enabling the generation of “armored” NK cells that resist immunosuppression within the TME. Examples include deletion of TGFBR2 to render NK cells resistant to TGF-β signaling, knockout of CISH to enhance IL-15 responsiveness and metabolic fitness, disruption of inhibitory receptors such as NKG2A or TIGIT, and engineering chemokine receptors including CXCR1, CXCR2, CXCR4, or CCR7 to improve tumor trafficking (An et al., 2026). Innovative synthetic biology approaches are incorporating logic-gated CAR circuits, cytokine-sensing transcriptional switches, and inducible activation systems that selectively enhance NK cell function within the tumor while minimizing off-target toxicity (Dong et al., 2026; Kim et al., 2026; Lee et al., 2026). *In vivo* NK cell engineering using viral vectors, lipid nanoparticles, extracellular vesicles, polymeric nanoparticles, or antibody-targeted nanocarriers offers additional opportunities to overcome poor trafficking and immune suppression, while preserving NK cell functionality within their native physiological environment. Local intratumoral delivery of mRNA or CRISPR payloads is under investigation to minimize systemic exposure while generating potent NK responses precisely where they are needed (Diwanji et al., 2024; Geng et al., 2026). Equally transformative is the improvement of iPSC-derived NK cells and their manufacturing at large scale with consistent phenotype and function, reduced batch-to-batch variability and facilitated multiplex gene engineering. The feasibility of producing highly engineered iPSC-NK products expressing CARs, membrane-bound cytokines, high-affinity CD16 variants and immune checkpoint modifications renders these universal platforms especially valuable for patients with rare cancers, where individualized manufacturing is often impractical. Most available evidence from mechanistic preclinical studies and early-phase clinical trials also suggests that combination therapies will likely become essential for achieving durable responses in solid tumors. Checkpoint inhibitors targeting PD-1/PD-L1, TIGIT, or NKG2A may reverse NK cell dysfunction, while mAbs can augment NK-mediated ADCC. Targeted therapies, radiation therapy, chemotherapy, and oncolytic viruses can further remodel the TME by increasing antigen expression, inducing immunogenic cell death, and promoting inflammatory cytokine release that enhances NK cell recruitment and activation. Cytokine agonists and NK cell engagers represent additional complementary strategies for maximizing antitumor responses (He et al., 2023; Liu et al., 2025).

Clinical development in rare cancers also faces unique logistical challenges beyond tumor biology. Extremely small patient populations severely limit the feasibility of conventional randomized clinical trials. Many ultra-rare malignancies lack appropriate representative animal models or patient-derived xenografts that recapitulate the human immune microenvironment. As a result, translational studies are underpowered, biomarker discovery is slow, and optimization of dosing, persistence, and combination strategies remains difficult. Future clinical development of NK cell therapies for rare and ultra-rare cancers will likely rely on adaptive platform trials, which use a single, ongoing master protocol to evaluate multiple therapies simultaneously. Within these platforms, basket designs can assess a therapy across multiple rare malignancies that share common molecular or immunologic features (Hirakawa et al., 2018; Renfro and Sargent, 2017). Biomarker-enriched enrollment can preferentially recruit patients whose tumors are most likely to respond based on predictive biomarkers, and seamless dose optimization can refine dose selection while transitioning efficiently from dose-finding to efficacy evaluation without requiring separate trials (Broglio et al., 2024; Hu and Dignam, 2019). Together, these approaches offer a more efficient and statistically robust framework for generating clinical evidence from the small patient populations typical of rare cancers (Krendyukov et al., 2021). For rare and ultra-rare solid malignancies, the future may lie less in disease-specific cell products and more in adaptable platform technologies capable of rapid customization. Universal CAR backbones, modular multispecific engagers, biomarker-driven patient selection, and collaborative basket clinical trials could accelerate clinical evaluation across multiple histologies that share common immunologic vulnerabilities.

## 7 Conclusion

Current evidence indicates that the use of allogeneic, off-the-shelf NK cell platforms might overcome several challenges relevant to rare and ultra-rare solid cancers. Cryopreserved NK cell products can be manufactured in advance, quality controlled, and administered without patient-specific production delays. This standardized manufacturing process reduces logistical complexity, facilitates multicenter trial participation, and enables rapid treatment of patients with aggressive diseases who may not be candidates for lengthy autologous manufacturing workflows. Moreover, because the same engineered NK cell product can be evaluated across multiple antigen-positive malignancies, off-the-shelf platforms offer a scalable development model that is particularly well suited to diseases with extremely limited patient populations.

Despite these advantages, important challenges remain, including improving NK cell persistence and trafficking to solid tumors, overcoming the immunosuppressive TME, identifying predictive biomarkers of response, and establishing durable clinical benefit in rigorously designed prospective studies. Addressing these barriers will require continued advances in NK cell engineering, rational combination strategies, and innovative clinical trial designs tailored to the realities of rare cancer research.

NK cell therapy has transformed from a promising experimental approach into a rapidly evolving therapeutic platform with broad translational potential. Although clinical evidence remains preliminary, the convergence of increasingly sophisticated cellular engineering, precision biomarker strategies, and efficient trial methodologies provides a realistic pathway to accelerate clinical development in rare and ultra-rare solid tumors. As these technologies mature, off-the-shelf NK cell therapies have the potential not only to expand access to cellular immunotherapy but also to redefine treatment paradigms for patients with rare malignancies, where effective therapeutic options remain critically limited. For patients with rare and ultra-rare solid tumors, where conventional therapeutic development has historically lagged, engineered NK-cell therapies offer the realistic possibility of bringing scalable, precision immunotherapy to diseases that have long lacked effective treatment options.
